# Saliva as Blood Alternative in Therapeutic Monitoring of Teriflunomide—Development and Validation of the Novel Analytical Method

**DOI:** 10.3390/ijms23179544

**Published:** 2022-08-23

**Authors:** Bartłomiej Sankowski, Sylwia Michorowska, Emilia Raćkowska, Mariusz Sikora, Joanna Giebułtowicz

**Affiliations:** 1 Department of Bioanalysis and Drug Analysis, Faculty of Pharmacy, Medical University of Warsaw, 02-097 Warsaw, Poland; 2 National Institute of Geriatrics, Rheumatology and Rehabilitation, Spartańska 1, 02-637 Warsaw, Poland

**Keywords:** therapeutic drug monitoring, teriflunomide, leflunomide, saliva, validation, LC-MS/MS

## Abstract

Therapeutic drug monitoring (TDM) is extremely helpful in individualizing dosage regimen of drugs with narrow therapeutic ranges. It may also be beneficial in the case of drugs characterized by serious side effects and marked interpatient pharmacokinetic variability observed with leflunomide and its biologically active metabolite, teriflunomide. One of the most popular matrices used for TDM is blood. A more readily accessible body fluid is saliva, which can be collected in a much safer way comparing to blood. This makes it especially advantageous alternative to blood during life-threatening SARS-CoV-2 pandemic. However, drug’s saliva concentration is not always a good representation of its blood concentration. The aim of this study was to verify whether saliva can be used in TDM of teriflunomide. We also developed and validated the first reliable and robust LC-MS/MS method for quantification of teriflunomide in saliva. Additionally, the effect of salivary flow and swab absorptive material from the collector device on teriflunomide concentration in saliva was evaluated. Good linear correlation was obtained between the concentration of teriflunomide in plasma and resting saliva (*p* < 0.000016, r = 0.88), and even better between plasma and the stimulated saliva concentrations (*p* < 0.000001, r = 0.95) confirming the effectiveness of this non-invasive method of teriflunomide’s TDM. The analyzed validation criteria were fulfilled. No significant influence of salivary flow (*p* = 0.198) or type of swab in the Salivette device on saliva’s teriflunomide concentration was detected. However, to reduce variability the use of stimulated saliva and synthetic swabs is advised.

## 1. Introduction

Therapeutic drug monitoring (TDM), which dates back to the 1970s, was initially used to determine drug’s therapeutic ranges [1], defined as the blood, serum or plasma concentrations expected to result in the desired therapeutic effects [2]. With time TDM was also found to be very useful for efficacy, compliance, drug–drug interactions assessment, toxicity avoidance as well as therapy cessation monitoring [1]. TDM may be, thus, very beneficial for optimizing therapy involving drugs characterized by serious side effects and marked interpatient pharmacokinetic variability such as leflunomide and its biologically active metabolite, teriflunomide, being orally administered immunomodulatory agents.

Leflunomide was first approved for the treatment of active rheumatoid arthritis (1998 by FDA (US Food and Drug Administration) and 1999 by the EU Commission) [3]. Indications were further extended to include active psoriatic arthritis (2004 by the EU Commission [4]). Preclinical studies have also reported anti-inflammatory [5], antineoplastic [5,6,7] and antiviral potential of leflunomide [8,9], as well as immunosuppression after organ transplantation [7,10]. However, so far, the drug has failed to receive approvals for these indications as some results seem to be contradictory or inconclusive [11,12]. Teriflunomide is approved for the treatment of multiple sclerosis (2010 by FDA [13] and 2013 by the EU Commission [14]).

Leflunomide, after being taken orally, is well absorbed and undergoes almost complete transformation (70–95%) into its long half-life (15.7 days) active metabolite teriflunomide during the first-pass intestinal and hepatic metabolism by cytochrome P450 (CYP) 1A2, CYP2C19 and CYP3A4 [12,15]. Teriflunomide selectively and reversibly inhibits an inner mitochondrial membrane enzyme dihydroorotate dehydrogenase (DHODH) [16]. DHODH is one of the enzymes required for the de novo biosynthesis of pyrimidine-based nucleotides which are essential to sustain proliferation of rapidly growing cells [17] including activated T and B lymphocytes without causing cell death [18].

Even though leflunomide is generally considered to be a safe drug [12], it is one of six disease-modifying anti-rheumatic drugs with the greatest number of reported adverse drug reactions [19] with the following ones being the most frequent: mild increase in blood pressure, leukopenia, paresthesia, headache, dizziness, diarrhea, nausea, vomiting, oral mucosal disorders, abdominal pain, increased hair loss, eczema, rash, pruritus, dry skin, tenosynovitis, anorexia, weight loss, asthenia, mild allergic reactions and elevation of liver parameters [20]. A serious disadvantage of leflunomide treatment is drug-induced hepatoxicity potentially leading to hepatitis and liver failure [21]. Moreover, because of its teratogenic effects, the drug is contradicted in pregnant women, and it is strongly advisable to avoid pregnancy before its complete elimination [22]. Taking into account the adverse effects of the two drugs, the time needed to reach steady-state therapeutic blood concentrations (around 2.5 months [12]) and marked interpatient variability in pharmacokinetics [23], their monitoring would be beneficial. Furthermore, non-invasive and effective monitoring of their concentrations in body fluids would allow to extend leflunomide/teriflunomide indications to include e.g., transplant recipients [10] that require higher doses [15]. Since leflunomide is almost completely metabolized to teriflunomide [12], the circulating levels of leflunomide are very low [24]. That is why TDM can be based on measuring the concentration of teriflunomide in either case.

Traditionally used TDM’s methods are based on the determination of drugs’ concentration in blood. However, blood collection has many disadvantages such as transient discomfort of patients, bruising, infections at the venipuncture site [25], poor patient compliance, high costs, potential excessive blood loss and the need of trained staff [26]. An appealing alternative to blood in terms of the quantification of drug’s concentration in patients is saliva.

Saliva is readily accessible and easily collectible [27] body fluid produced by three paired major salivary glands and numerous minor salivary glands present in the oral mucosa [28]. However, some components of saliva may be also derived from the blood by passive diffusion, active transport, ultrafiltration or pinocytosis [27]. Drugs in most cases enter the saliva by passive diffusion and their salivary concentrations reflect the free fraction of the drug in blood corresponding to the pharmacologically active fraction being in equilibrium with the protein-bound fraction [27]. This makes saliva an attractive diagnostic material for e.g., drug monitoring [27], especially in the case of highly protein-bound drugs such as teriflunomide [12,29]. Sampling of saliva is noninvasive, painless, poses lower risk of infection and does not require trained personnel. Consequently, it is much more suitable for repeated sample collection, even in children, which can be easily performed at home [26].

Nowadays, it is more and more common to replace blood with other matrices in TDM. The applicability of saliva for this purpose has become increasingly important as evidenced by the numerous research projects, most of which found good correlation between blood and saliva concentration of antiepileptic [30,31,32], immunosuppressive [29,33,34,35] or antitubercular drugs [36]. However, it should be noted that drug penetration into saliva (drug distribution from blood to saliva via passive diffusion) is affected by several factors, the most important being salivary pH and flow rate as well as drug’s stability in a given matrix [36]. Therefore, the effect of these factors on the drug’s salivary concentration should be evaluated during method validation.

Since all therapeutic activity of leflunomide is due to teriflunomide, there are several validated analytical methods available in literature to quantify teriflunomide in traditionally used matrices such as plasma, serum or whole blood [15]. These include HPLC-UV [37,38,39,40], strong cation exchange HPLC [41] and LC-MS/MS [40,42,43,44,45,46]. LC-MS/MS was also successfully applied to quantify teriflunomide in urine [46]. However, to date there is no method available for the determination of salivary concentrations of this compound. Moreover, it should be evaluated if there is a tight correlation between the saliva and blood concentrations of teriflunomide to confirm if saliva could be useful for quantitative predictions of its plasma levels, and consequently for TDM.

The aim of this study was to verify whether saliva can be used in TDM of teriflunomide. We also developed and validated the first reliable and robust LC-MS/MS method for the quantification of teriflunomide in saliva. Additionally, the effect of salivary flow and swab absorptive material from the collector device on teriflunomide concentration in saliva was evaluated.

## 2. Results and Discussion

### 2.1. Application of the Salivette Device

Commercially available devices made of absorptive materials are the most popular methods of collecting saliva as they are considered more hygienic and easier to handle than passive drool [47]. Despite their popularity they are rarely validated for a specific analyte collection (e.g., Salivette Cortisol from Starstedt [48]), which potentially leads to biased results as some of the collection swabs are known to adsorb the analyte due to unspecific binding [47]. This is the case with Δ9-tetrahydrocannabinol (15–20% loss with Quantisal collector (cellulose pad [49]) and 20–79% loss with the Certus device (polyethylene pad [49])) [50], methadone (recovery of 28% using Certus collector) [49], 2-ethylidene-1,5-dimethyl-3,3-diphenylpyrrolidene (major metabolite of methadone, 45% using a DCD5000 Device (the material of the pad cannot be identified based on available literature)) [51], as well as melatonin, insulin and interleukin-8 (recoveries < 32% using cotton Salivette) [52]. The best recoveries are usually achieved with synthetic Salivettes [52] or Porex Saliva Collection Swab and Drager DCD^TM^5000 [47]. The low performance of cotton swabs is partially due to cotton-derived substances interfering with immunoassays as well as due to the adsorption of analytes on the cotton [52]. Low-density polyethylene Certus collectors are known to adsorb compounds with long lipophilic chains such as THC [49]. No device is suitable for all analytes therefore their interference with the collection device should be evaluated before their quantification to ensure reliable results. The most popular swabs used in Poland are cotton and synthetic Salivettes. Taking into account advantages of synthetic swabs over cotton ones, we assumed good performance of the first mentioned type of Salivette in our studies, which was confirmed by recovery experiments.

The recovery of teriflunomide from the cotton swab was 94% (RSD = 5.6%), whereas recovery from the synthetic swab was 102% (RSD = 8.8%). The results suggest that none of the analyzed swabs had a significant influence on teriflunomide concentration in saliva.

### 2.2. Method Validation

The retention time of teriflunomide and its internal standard was 4.8 min.

#### 2.2.1. Linearity and Selectivity

The calibration curve obtained by weighted linear regression analysis was linear in the range 2–500 ng mL^−1^ regarding the peak area ratio of teriflunomide and the internal standard versus the nominal concentration of teriflunomide. The values of regression parameters for the curve, described by the equation: y = ax + b, were calculated as: a = 0.0029 (SD = 0.0009), b = 0.0183 (SD = 0.0069) and R^2^ = 0.999 (0.9988–0.9998). All regression parameters were statistically significant (*p* < 0.05).

**Figure 1:**
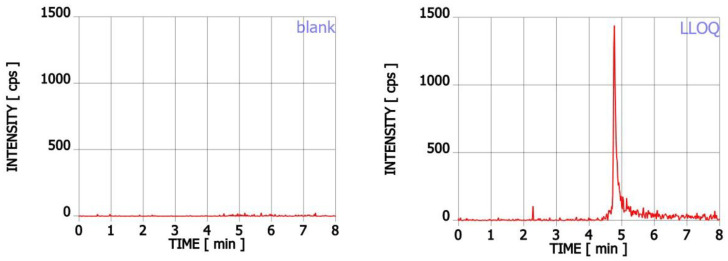
The chromatograms obtained for blank sample and saliva spiked with teriflunomide at LLOQ level (2 ng mL^−1^).

The method was selective. The chromatograms obtained for blank saliva sample and saliva spiked with teriflunomide at LLOQ level (2 ng mL^−1^) are presented in Figure 1.

#### 2.2.2. Accuracy and Precision

The accuracy and precision for LLOQ and QC samples within one day (*n* = 5) and between runs (*n* = 15) met the acceptance criteria (Table 1).

The carry-over experiment, in which blank samples were analyzed just after the highest concentration calibration standards, did not show any signals influencing quantification.

#### 2.2.3. Matrix Effect and Recovery

The matrix factors for different lots of saliva for teriflunomide and the internal standard were 98% and 97% for the low concentration, 101% and 103% for the medium concentration and 99% and 99% for the high concentration, respectively. The CV of the IS-normalized matrix factor was 2.1% for 10 ng mL^−1^, 1.8% for 250 ng mL^−1^ and 1.8% for 450 ng mL^−1^, respectively.

Method recovery of the analyte for 10 ng mL^−1^ was 94% (CV = 6%), for 250 ng mL^−1^ it was 97% (CV = 6%), whereas for 450 ng mL^−1^ it was 104% (CV = 5%). In the case of IS, recovery was between 99–103% and CV under 2% for each sample series.

#### 2.2.4. Stability and Dilution Integrity

The freeze/thaw stability (*n* = 10), short- (*n* = 5) and long-term (*n* = 5) stability in the matrix, and stability in the autosampler after 24 h (*n* = 5) and 48 h (*n* = 5) met the acceptance criteria (Table 2).

Stock solution stability at different temperatures ranged from 98 to 102% (RSD = 1.6–4.1%). Dilution integrity test also fulfilled the validation criteria (RSD = 4.6%, accuracy = 99%).

### 2.3. Influence of Salivary Flow

**Figure 2:**
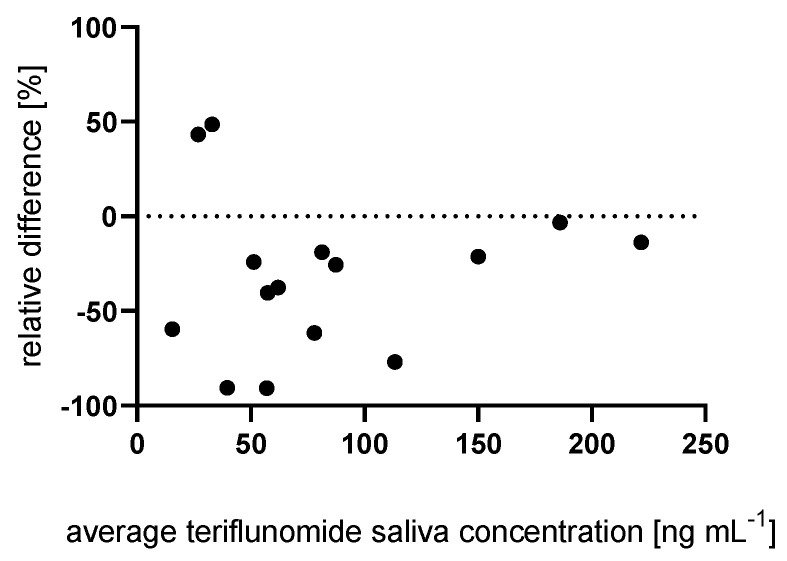
Relative difference between teriflunomide concentration in resting and stimulated saliva collected using the Salivette with synthetic swab. Relative difference was calculated using the following equation: [(resting saliva concentration – stimulated saliva concentration) × (average of resting and stimulated saliva concentration ${)}^{-1}$ ] × 100%.

The mean concentrations of teriflunomide in resting and in stimulated saliva were 72 ng mL^−1^ (SD = 58) and 96 ng mL^−1^ (SD = 64), respectively. Statistical analysis revealed no significant difference in the concentration of teriflunomide (*p* = 0.198) between resting and stimulated saliva. However, in some patients these differences were high (Figure 2). Therefore, only one type of saliva should be used in clinics.

Several factors affect salivary secretion and saliva’s composition. Stimulation usually changes the pH [53] by increasing the excretion of bicarbonate [36] leading to significantly, but only slightly higher values [54], which in turn affects salivary concentration of a drug. Changes in pH can affect the passage of ionizable substances from blood to saliva and the other way around. However, this applies only to those compounds for which the ratio of ionizable: non-ionizable form changes within pH range. Teriflunomide (pKa of 5.48 [55]) is ionized in a very broad pH range, therefore the changes in slightly acidic salivary pH did not affect its concentration in saliva.

Stimulated saliva has many advantages over the resting one. Upon stimulation, larger volumes of less viscous saliva can be obtained in shorter period of time, there is lower pH gradient between plasma and saliva and the saliva/plasma concentration ratios are more consistent for some drugs [53], therefore stimulated saliva may be recommended for the teriflunomide quantification.

### 2.4. Correlation between the Plasma and Saliva Concentration of Teriflunomide

**Figure 3:**
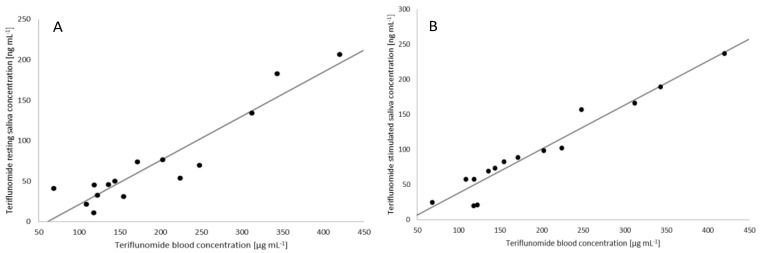
The correlation between teriflunomide concentration in blood (${\mu g\;\mathrm{mL}}^{-1}$) and either resting (**A**) (${\mathrm{ng}\;\mathrm{mL}}^{-1}$ ) or stimulated (**B**) (${\mathrm{ng}\;\mathrm{mL}}^{-1}$ ) saliva.

A good linear correlation was obtained between the concentration of teriflunomide in plasma and resting saliva (*p* < 0.000016, r = 0.88) (Figure 3), and even better between plasma’s and stimulated saliva’s concentrations (*p* < 0.000001, r = 0.95). The concentration in stimulated saliva was well correlated with the concentration in resting saliva (*p* < 0.000011, r = 0.89).

The correlations between the concentration of teriflunomide in plasma and either resting or stimulated saliva (r = 0.88, r = 0.95, respectively) determined in this study are comparable with correlations determined for other drugs such as carbamazepine (r = 0.91–0.99), phenytoin (r = 0.89–0.98) or phenobarbital (r = 0.92–0.96) [53]. They are stronger than that between the LC-MS/MS-determined cortisol concentrations in serum and saliva (r = 0.75) [56] used in clinically accepted non-invasive functional stress assay. Our results suggest that LC-MS/MS quantification of teriflunomide’s concentrations in saliva is a reliable, sensitive and non-invasive method which could be applied in clinical settings for therapeutic teriflunomide monitoring. Importantly, no adverse events related to the method of saliva collection were reported.

## 3. Materials and Methods

### 3.1. Chemicals

Reference standard teriflunomide and internal standard teriflunomide-d4 were purchased from Toronto Research Chemicals (TRC, Toronto, ON, Canada). Solvents, HPLC gradient grade methanol, acetonitrile, DMSO and formic acid 98% were purchased from Merck (Darmstadt, Germany). Ammonium acetate was purchased from Avantor Performance Materials (Gliwice, Poland). Ultrapure water was obtained from a Millipore water purification system (Milli-Q, Billerica, MA, USA).

### 3.2. Standard Solutions, Calibration Standards and Quality Control Samples

The stock solutions of the analyzed compounds, teriflunomide and teriflunomide-d4, were prepared by weighing of the appropriate mass of each compound and dissolving it in DMSO to obtain a concentration of 5 mg mL^−1^ and 1 mg mL^−1^, respectively. The working standard solutions were prepared prior to use by dilution of the appropriate stock solutions with ultrapure water to obtain the required concentrations. All stock solutions were stored at −20 °C. The calibration standards for teriflunomide were made at concentrations 2–500 ng mL^−1^ after saliva enrichment. The quality control (QC) samples were prepared using blank saliva and the following levels of teriflunomide: 10 ng mL^−1^ (QClow), 250 ng mL^−1^ (QCmedium) and 450 ng mL^−1^ (QChigh). The calibration standards and QC samples were stored at −26 °C until required.

### 3.3. Clinical Samples

Fifteen patients from rheumatology clinics at the National Institute of Geriatrics, Rheumatology and Rehabilitation (Warsaw, Poland) were recruited after giving informed consent. All patients were on immunosuppressive therapy taking 15–20 mg of leflunomide daily for rheumatoid arthritis or psoriatic arthritis. Patients were excluded if they had: active lesions of the oral mucosa, Sjögren’s syndrome or any other disease associated with a decrease in the secretion of saliva, abnormal renal and/or liver function. The sample consisted of 10 women (67%) and 5 men (33%), aged 28–67 years (median = 48 years, interquartile range (IQR) = 13 years). Matching blood and saliva samples were collected at the same time in the fasted state between 8:00 a.m. and 11:00 a.m. All participants abstained from food, beverages, other drugs intake, brushing teeth, chewing gum and smoking for at least 8 h before collection. Blood was collected with ethylenediaminetetraacetic acid as anticoagulant, thoroughly mixed and centrifugated at 425 g for 20 min to obtain plasma, which was stored at −80 °C until further analyses. Resting and stimulated saliva samples were collected using synthetic swabs (Salivette system, Sarstedt, Nümbrecht, Germany) using modified manufacturer’s procedure by a single examiner. Patients were asked to sit in an upright position on a chair with their head tilted slightly forward. Prior to saliva sampling, the volunteers rinsed their mouth with room temperature water and rested in a quiet room. After about 5 min, the synthetic swab from the Salivette device was put under the tongue for 5 min (resting saliva). Volunteers were asked to avoid swallowing and oral movements during collection. If the swab did not absorb enough saliva, the collection time was extended. Next, patients were asked to rinse their mouth again with room temperature water and then to chew another swab for 1–2 min to collect stimulated saliva. All swabs were transferred into test tubes and centrifuged at 945× *g* at 20 °C for 3 min to obtain saliva samples. Next, obtained saliva samples were stored at −80 °C till the analysis.

### 3.4. Sample Extraction

Thawed plasma samples were first diluted 300 times then vortexed for 1 min, mixed with internal standard solution (1:10, *v*/*v*), deproteinized with acetonitrile (1:5, *v*/*v*), vortexed for 1 min and centrifuged at 10,500× *g* at 4 °C for 10 min. The supernatant was diluted 1:1 (*v*/*v*) with ultrapure water and injected onto the column.

On the day of analysis, the thawed saliva samples were vortex for 1 min, mixed with internal standard solution (1:10, *v*/*v*), deproteinized with acetonitrile (1:4, *v*/*v*) incubated at −20 °C for 20 min and centrifuged at 10,500× *g* at 4 °C for 10 min. The supernatant was injected onto the column.

### 3.5. Chromatographic and Mass Spectrometric Conditions

Instrumental analysis was performed using an Agilent 1260 Infinity (Agilent Technologies, Santa Clara, CA, USA) equipped with a degasser, autosampler and binary pump, coupled to a hybrid triple quadrupole/linear ion trap mass spectrometer QTRAP 4000 (AB Sciex, Framingham, MA, USA). The curtain gas, ion source gas 1, ion source gas 2 and collision gas (all high purity nitrogen) were set at 35 psi, 60 psi, 40 psi and ‘medium’ instrument units, respectively. The ion spray voltage and source temperature were set at 4500 V and 600 °C, respectively. Chromatographic separation was achieved with a Kinetex C-18 column (100 mm, 4.6 mm, particle size 2.6 µm) supplied by Phenomenex (Torrance, CA, USA). The column was maintained at 40 °C at a flow rate of 0.5 mL min^−1^. The mobile phases consisted of 2.5 mM solution of ammonium acetate with 0.2% formic acid as eluent A and acetonitrile with 0.2% formic acid as eluent B. The gradient (B) was as follows: 0 min 30%; 1 min 30%; 3 min 95%; 9 min 95%; 9.1 min 30%; 12 min 30%. The injection volume was 10 µL. The target compounds were analyzed in multiple reaction monitoring (MRM) mode, monitoring two transitions between the precursor ion and the most abundant fragment ions for each compound. The transitions used for the quantification were m/z 268.9 > 81.9 and m/z 272.9 > 81.9 for teriflunomide and teriflunomide-d4, respectively. The compound parameters, i.e., declustering potential (DP), collision energy (CE), entrance potential (EP) and collision exit potential (CXP), were −60, −30, −10 and −13 V and −70, 30, −10 and −7 V for teriflunomide and teriflunomide-d4, respectively.

### 3.6. Application of the Salivette Device

Assessment of the utility of the Salivette device in saliva collection for teriflunomide quantification was performed. To estimate the potential retention of the compound on the swabs, the cotton swabs (from Salivette) and synthetic swabs (from Salivette cortisol) were incubated in pooled saliva samples of known teriflunomide concentration (QCmedium). The incubation was performed for 3 min at 4 °C. The swabs were placed back in the Salivette devices and centrifuged at 945× *g* at 20 °C for 3 min. The concentration of teriflunomide in the samples was compared with its concentration in saliva samples incubated without swabs. Swab recoveries within the range of 85–115% were considered acceptable.

### 3.7. Method Validation

Material for validation experiments was the pooled saliva of healthy volunteers. The validation was performed according to the EMA (European Medicines Agency) [57] and FDA [58] guidelines. Briefly, the linearity range was selected as 2–500 ng mL^−1^ of teriflunomide. Calibration curves were prepared in quintuple. The accuracy and precision of the method were determined within run and between run using a lower limit of quantification (LLOQ) and QC samples (10, 250 and 450 ng mL^−1^) for teriflunomide. Acceptance criteria were ≤15% (≤20% for LLQ) for precision and 85–115% (80–120% for LLQ) for accuracy. Carry over was also studied.

The samples for matrix effect (calculated as matrix factor, MF) [57] were prepared from blank saliva. MF was calculated as the ratio of the instrument response to teriflunomide with IS in extracted saliva spiked post-extraction and teriflunomide with IS in pure solvent at three teriflunomide concentrations (QClow, QCmedium and QChigh) in saliva of six different sources. The CV of the IS-normalized matrix factor should not exceed 15%.

The recovery was calculated as the ratio of the instrument response to teriflunomide (or IS) in extracted saliva spiked before and after extraction, and at three teriflunomide concentrations (QClow, QCmedium and QChigh) in saliva of six different sources.

The stability of teriflunomide was confirmed under various conditions using QC samples (QClow and QChigh). The tests included: freeze/thaw stability (three cycles), short- (4 h at 24 °C) and long-term (24 h at 24 °C) stability of the analyte in the matrix, stability in the autosampler (24 h and 48 h at 4 °C) and stock solution stability at different temperatures (−26 °C, 30 days; 4 °C, 7 days; room temperature, 1 day). The accepted values for stability and accuracy are 85–115%.

Dilution integrity was tested by spiking the blank saliva with an analyte concentration five times greater than the highest concentration used to prepare the calibration curve and diluting this sample with a blank saliva (*n* = 5). Accuracy should be within 85–115% and precision within ±15%.

The method of teriflunomide determination in plasma was validated previously (data not published). Briefly, the linearity was in the range of 1.5–300 µg mL^−1^ (R^2^ = 0.986–1.000). The method’s within-run accuracy was 105–109% (CV = 2–6%) for LLOQ (1.5 µg mL^−1^), 103–109% (CV = 3–5%) for 3 µg mL^−1^, 96–104% (CV = 2–4%) for 150 µg mL^−1^ and 91–106% (CV = 1–3%) for 262.5 µg mL^−1^ of teriflunomide in plasma. The between-run accuracy was 107% (CV = 5%), 106% (CV = 4%), 99% (CV = 5%) and 97% (CV = 7%) for the concentration of 1.5 µg mL^−1^, 3 µg mL^−1^, 150 µg mL^−1^ and 262.5 µg mL^−1^, respectively. The matrix factors for different lots of plasma for teriflunomide and the internal standard were 104% and 102% for the low concentration (3 µg mL^−1^), 103% and 102% for the high concentration (262.5 µg mL^−1^), respectively. The CV of the IS-normalized matrix factor was 3% for 3 µg mL^−1^, and 4% for 262.5 µg mL^−1^. Method recovery of the analyte was 102% for both concentrations (i.e., 3 µg mL^−1^, 262.5 µg mL^−1^). In the case of IS recovery was between 106–108%.

### 3.8. Statistical Analysis

The statistical evaluation of the results was performed with STATISTICA software 12.0 (StatSoft Polska, Kraków, Poland) for Windows licensed to the Medical University of Warsaw. The Shapiro–Wilk test was used to evaluate the normal distribution of the results. The Wilcoxon test was used to evaluate the influence of stimulation on teriflunomide concentration in saliva. Pearson’s correlation coefficient was used to measure the statistical association, between two continuous variables.

## 4. Conclusions

Saliva can be used as an alternative matrix to blood in TDM of teriflunomide. Stimulated saliva collection with the synthetic swab is recommended. The proposed LC-MS/MS method can be used for that purpose due to being robust, accurate and sensitive.

Even though the obtained results are promising this study has some limitations. First of all, the study population consisting of 15 patients was relatively small. While diagnostic utility of saliva drug monitoring may be influenced by several conditions and pathologies, we excluded patients with oral bleeding or inadequate saliva production. Additionally, all samples were collected at fasting and after restraint from smoking and oral hygiene procedures, to minimize the risk for contamination. Drug salivary concentration in patients with inflammatory rheumatic disorders should be interpreted with caution due to more common prevalence of salivary glands exocrine dysfunction (leading to poor specimen yield) and periodontal disease (leading to contamination with blood). The latter, in particular, can strongly affect the level of the drug in saliva. Therefore, further studies are required to evaluate the clinical significance of teriflunomide saliva concentration on patient outcomes.

Taking all of the above together, despite the limitations listed, the validated method described in this study allows for the practical use of good-quality saliva in TDM of teriflunomide. However, further studies on larger population are needed.

## Figures and Tables

**Table 1:** Precision and accuracy data for teriflunomide determination in salvia.

Nominal Concentration (ng mL^−1^)	2 (LLOQ)	10 (QClow)	250 (QCmedium)	450 (QChigh)
Within-run precision (%) (*n* = 5)	3.0–9.0 ^a^	2.0–7.0	3.0–12	4.0–9.0
Within-run accuracy (%) (*n* = 5)	101 ^b^	109	104	104
Between-run precision (%) (*n* = 15)	12 ^a^	7.0	9.0	7.0
Between-run accuracy (%) (*n* = 15)	112 ^b^	105	96	92

Accepted precision: ≤15% (^a^ ≤20%). Accepted accuracy: 85–115% (^b^ 80–120%).

**Table 2:** Stability data of teriflunomide in saliva (short- and long-term) as well as in the extract in autosampler.

Stability Test (%)		10 ng mL^−1^		450 ng mL^−1^	
		Stability	Accuracy	Stability	Accuracy
Freeze/thaw stability		95	113	94	95
Stability in autosampler	after 24 h	104	103	100	92
	after 48 h	102	100	100	93
Short-term stability		102	101	97	107
Long-term stability		91	104	103	101

Stability (accepted value: 85–115%); Accuracy (accepted value: 85–115%).

## Data Availability

The data are available from the Authors on request.
